# Coronal and sagittal spinal alignment in lumbar disc herniation with scoliosis and trunk shift

**DOI:** 10.1186/s13018-019-1300-0

**Published:** 2019-08-20

**Authors:** Weifei Wu, Ying Chen, Ling Yu, Fei Li, Weichun Guo

**Affiliations:** 1 Department of Orthopedics, Renmin Hospital of Wuhan University, Wuhan, Hubei China; 2 Department of Nephrology, The People’s Hospital of Three Gorges University, the First People’s Hospital of Yichang, Yichang, Hubei China

## Abstract

**Background:**

To investigate the incidence of scoliosis and trunk shift in patients with LDH (lumbar disc herniation) and analyze the differences in spinopelvic alignment among patients with or without trunk shift and non-symptom controls.

**Materials and methods:**

All included subjects had standard upright antero-posterior and lateral radiographs of the whole spine taken. Evidence of disc herniation was confirmed by computed tomography or magnetic resonance imaging. The parameters measured included trunk shift and Cobb angle, TK (thoracic kyphosis), TLK (thoraco-lumbar junction kyphosis), LL (lumbar lordosis), PI (pelvic incidence), SS (sacral slope), PT (pelvic tilt) and SVA (sagittal vertical axis).

**Results:**

Sixty-eight patients with LDH and 61 controls were included. There were significantly more male patients with trunk shift than the patients without trunk shift. Forty-two patients had curve magnitudes ranging from 5 to 38°. The trunk shift ranged from 0.5 to 7.3 cm. A total of 54.76% of patients had a disc herniation on the concave side of the main curve. Fifty percent of patients showed a trunk shift towards the opposite side of disc herniation. There were significant differences in spinopelvic parameters among groups. Significant correlations were also observed between several spinopelvic parameters in the three groups. However, the degrees of correlations among the spinopelvic parameters differed among the three groups.

**Conclusion:**

Spinal sagittal morphology in LDH patients with trunk shift exhibits a more anterior shift of the C7 plumb line, less LL, and a more horizontal sacrum. Correlation analysis indicated a disharmonious spinopelvic interaction and a change in the compensatory model in patients with LDH.

## Background

Lumbar disc herniation (LDH) is becoming increasingly common based on population-based studies. Patients with LDH may complain of paravertebral muscle pain, leg pain, walking difficulty, scoliosis, trunk shift and spinal sagittal alignment imbalance, which produce many problems to both individuals and society [1, 2]. Scoliosis and trunk shift have been found in both adolescent and adult patients with LDH [3, 4]. Although the pathophysiology is not adequately comprehended, scoliosis and trunk shift have been seen as compensatory behaviour of the body to ease the stimulation of nerves. Spinopelvic imbalance was found in patients with LDH who have symptoms [5–9]. The development of these symptoms could result in certain alterations in the sagittal and coronal shape of the spine. In addition, abnormalities of sagittal spinal alignment and pelvis morphology can cause these symptoms to be more serious. To our knowledge, although there have been a few studies about spinopelvic balance and the relationship among those parameters in patients with LDH, no study focussed on the differences in spinopelvic alignment between patients with/without scoliosis or trunk shift caused by LDH has been reported. The present study prospectively studied the incidence of scoliosis and trunk shift in patients with LDH and analyzed the differences of spinopelvic alignment among patients with or without trunk shift and non-symptom controls, aiming to study the sagittal alignment changes in LDH patients with and without trunk shift in the coronal plane.

## Materials and methods

### Healthy subjects and patients

The review board of the Renmin Hospital of Wuhan University approved the entire research plan before the present study. Adult volunteers were recruited from the medical examination centre of our hospital during a periodic health screening scheme. Inclusion criteria included age (20–75 years), no leg and back pain caused by LDH, no previous spinal-related surgery and no history of trauma and other local problems. Patients with LDH at orthopaedic outpatient service and the inpatient department were included from September 2016 to August 2017. Patients with LDH who were aged 20–75 years and had evidence of LDH verified by magnetic resonance imaging (MRI), neurological symptoms, no previous spine surgery and injury and no definite history of scoliosis were included in the study. Exclusion criteria included adolescent or adult idiopathic scoliosis with LDH, adolescent with LDH, spondylolisthesis, lumbar instability and spinal stenosis. These volunteers and patients signed an informed consent that allowed their clinical data to be used for the research study. These subjects were examined by two orthopaedic surgeons, and then standard upright lateral and antero-posterior radiographs of the whole spine were taken.

### Radiographic measurements

**Fig. 1:**
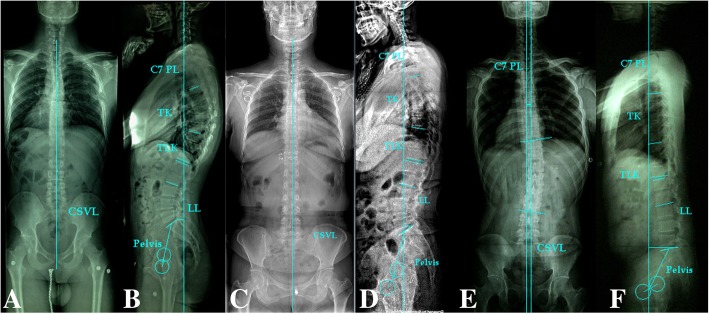
The examples of spinopelvic morphology in control, LDH patient without trunk shift and LDH patient with trunk shift. **a**, **c**, **e** The feature of the coronal plane in control (male; without trunk shift), LDH patient without trunk shift (male, L5/S1disc herniation) and LDH patient with trunk shift (male, L5/S1disc herniation; left shift: 0.8 cm, right thoracolumbar curve: 18°), respectively. **b**, **d**, **f** The measurement of spinopelvic parameters. TK thoracic kyphosis, TLK thoraco-lumbar junction kyphosis, LL lumbar lordosis, PI pelvic incidence, SS sacral slope, PT pelvic tilt, SVA sagittal vertical axis, CSVL central sacral vertical line. The parameters are showing as follows:

Curve characteristics, including curve magnitude, levels, direction and patterns, were estimated on the antero-posterior radiographs. The Cobb angle method was used to measure the magnitude of curvature. With regard to a patient with double curves, the curve with the maximum Cobb angle was selected as the main curve for analyzing the correlation between curve direction and LDH direction. If the apex of the curve is above the T8 vertebrae, a patient or control adult was considered normal in the coronal plane, as shown in Fig. 1. The trunk shift was the horizontal distance between the central sacral vertical line (CSVL) and the C7 plumb line (PL). In addition, the C7PL shift to the left of the CSVL was defined as a negative balance, and the C7PL shift to the right was defined as a positive balance. Trunk shift values greater than 2.0 cm were considered poor balance or decompensation in the coronal plane [10]. On upright lateral radiographs, the measured radiographic parameters included lumbar lordosis (LL), thoraco-lumbar junction kyphosis (TLK), thoracic kyphosis (TK), sacral slope (SS), pelvic incidence (PI), pelvic tilt (PT) and sagittal vertical axis (SVA) from C7 PL. TK was defined as the angle between the T12 inferior end plate and the T5 superior end plate. LL was defined as the angle between the L1 plate and the S1 superior end plate. TLK was measured by the T10 superior end plate and L2 inferior end plate. PI, which is a morphologic parameter, is the angle between the vertical line to the upper sacral endplate at its midpoint and the line linking this point with the femoral head axis. The SS is an angle between the horizontal and the upper sacral endplate. The PT is measured between the line through the midpoint of the sacral plate to the femoral head axis and the vertical head axis. The SVA was measured by the horizontal distance from the S1 postero-superior corner to the C7 PL. If C7PL was behind the postero-superior corner of S1, SVA was considered negative. If C7PL was ahead of the postero-superior corner of S1, SVA was considered positive. The level and direction of LDH were determined according to CT or MRI.

**Table:** 

Image	PI°	PT°	SS°	LL°	TLK°	TK°	SVA (cm)	Age (years)
B	47	10	37	60	5	40	-2.2	25
D	47	15	32	44	3	19	1.0	32
F	36	25	11	0	-8	-5	6.2	34

### Statistical analysis

Patients with LDH were divided into two groups depending on whether they were accompanied by trunk shift as well as LDH with shift (group A) and without shift (group B). One-way analysis of variance (ANOVA) was performed to analyze the differences in spinopelvic parameters among group A, group B and the control group. The gender difference between groups A and B was analyzed by Fisher’s exact test. The differences between any two groups among the three groups in the ANOVA were assessed further by Tukey’s studentized range test. In addition, the chi-square test was performed to analyze the relationship between the side of LDH and the direction of trunk shift/the direction of curve in the coronal plane and the relationship between levels of LDH and the direction of trunk shift. Pearson’s correlation coefficient was used to analyze the associations between two variables. *P* values (≤ 0.05) were considered statistically significant.

## Results

### Intergroup comparison of measured variables

A total of 68 patients with LDH and 61 controls were included. Fisher’s exact test (*P* = 0.018) showed that there were more male patients in group A than in group B. As shown in Fig. 1 and Table 1, a smaller age, PI, SS, LL, TLK and TK, and larger PT and SVA in group A were found when compared to the normal subjects. Significant differences were found between the two groups with regard to the PT, SS, LL, TLK, TK and SVA absolute values. In group B, no significant difference was found in PI, PT, SS and age compared with the control group, and LL, TLK and TK were significantly lower than those of the control group. When group A was compared with group B, no significant difference in all spinopelvic parameters and age except SVA absolute value, which was notably lower, and LL, which was notably higher, in group B was found.

**Table 1:** Differences in spinopelvic parameters between the patients with trunk shift and controls and the patients without trunk shift and controls

LDH patients	PI°	PT°	SS°	LL°	TLK°	TK°	SVA (mm)	Age (years)
With trunk shift	43.69 (9.13)	21.24^£^ (8.66)	22.45^§^ (8.06)	22.45^¢^ (17.78)	0.05^¥^ (7.67)	11.69^$^ (11.84)	48.36^δ^ (45.40)	38.86 (13.72)
Non-trunk shift	47.27 (7.61)	19.08 (8.56)	28.19 (8.64)	38.12^¢^ (13.09)	2.231^μ^ (7.50)	15.96* (10.48)	16.86^δ^ (29.59)	41.69 (11.55)
Controls	43.79 (12.32)	15.52^£^ (6.14)	28.26^§^ (11.44)	50.02^¢^ (8.50)	7.87^¥μ^ (7.02)	32.77*^$^ (8.93)	− 7.45^δ^ (31.56)	36.89 (12.44)
*P* value	0.316	0.000	0.010	0.000	0.000	0.000	0.000	0.268

For each parameter, mean and SD are showed

*LDH* lumbar disc herniation, *TK* thoracic kyphosis, *TLK* thoraco-lumbar junction kyphosis, *LL* lumbar lordosis, *PI* pelvic incidence, *SS* sacral slope, *PT* pelvic tilt, *SVA* sagittal vertical axis

“£,” “§,” “¥,” “μ,” “*” and “$” indicate significant difference between two groups (*P* < 0.05), and “¢” and “δ” indicate significant difference between any two groups among the three groups (*P* < 0.05)

As for the curve, 42 of 68 patients (61.8%) had curve aptitude ranging from 5 to 38° (average, 16.5°). Twenty-two patients (52.4%) had a thoracic curve, 10 patients (23.8%) had a thoracolumbar curve, 8 patients (19.0%) had a lumbar curve and 2 patients (4.8%) had a double curve. The trunk shift was observed from 0.5 to 7.3 cm (mean, 2.6 cm) in 42 of 68 patients (61.8%). Twenty patients (29.4%) showed a poor coronal balance because a trunk shift was more than 2.0 cm away from the midline. Thirty patients had a left-sided disc herniation, 27 patients had a disc herniation on the right side, and 11 patients had double-sided disc herniation. The location of DH at the concave side of the main curve was found in 54.76% (23/42) of patients (Table 2). A significant association was found between the side of the DH and the direction of the curve (*P* = 0.026). A trunk shift towards the opposite side of DH was noted in 50% (21/42) of patients (Table 3), and no notable correlation between the side of DH and the direction of trunk shift was observed (*P* = 0.215). There was no significant difference on the side of DH between group A and group B (*P* = 0.308) (Table 4).

**Table 2:** Association between the side of main curve and the side of disc herniation

Side of scoliosis	Side of disc herniation			
	Right	Left	Bilateral	Total
Right	4	12	2	18
Left	11	6	7	24
Total	15	18	9	42

*P* = 0.026

**Table 3:** Association between the direction of trunk shift and the side of disc herniation

Direction of trunk shift	Side of disc herniation			
	Left	Right	Bilateral	Total
Right	10	4	3	17
Left	8	11	6	25
Total	18	15	9	42

*P* = 0.215

**Table 4:** Differences in side of disc herniation between the patients with trunk shift and without trunk shift

LDH patients	Side of disc herniation			
	Left	Right	Bilateral	Total
With trunk shift	18	15	9	42
Non- trunk shift	12	12	2	26
Total	30	27	11	68

*P* = 0.308

### Correlations between measured variables

There were significant correlations between several spinopelvic parameters among the three groups (Table 5). The degrees of correlation varied among the three groups. PI was more strongly correlated with PT (*r* = 0.591/0.387) and less strongly with SS (*r* = 0.499/0.866) and LL (*r* = 0.283/0.582) when group A was compared to the control group. PT was more seriously negatively correlated with TK (*r* = − 0.419/0.274) and SS (*r* = − 0.405/− 0.120). SS was more strongly positively correlated with TK(*r* = 0.661/0.169) and LL (*r* = 0.855/0.613). LL was more notably correlated with TK (*r* = 0.770/0.209) and SVA (*r* = -0.646/0.111). When group B was compared with the control group, PT was more negatively correlated with TK(*r* = − 0.458), SS (*r* = − 0.608) and LL (*r* = − 0.671). TK was more strongly positively correlated with LL(*r* = 0.721) and SS (*r* = 0.534). SS was more strongly positively correlated with LL (*r* = 0.925).

**Table 5:** Correlations between several spinopelvic parameters in the three groups

LDH patients	With non- trunk shift		With trunk shift		Controls	
	*r*	*P* value	*r*	*P* value	*r*	*P* value
PI-PT	0.434	*0.027*	0.591	*0.000*	0.387	*0.002*
PI-SS	0.451	*0.021*	0.499	*0.001*	0.866	*0.000*
PI-LL	0.295	0.143	0.283	0.069	0.582	*0.000*
PI-TLK	0.382	0.054	0.384	*0.012*	0.011	0.934
PI-TK	0.091	0.657	0.186	0.238	0.294	*0.022*
PI-SVA	− 0.040	0.845	− 0.090	0.573	0.204	0.114
PT-SS	− 0.608	*0.001*	− 0.405	*0.008*	− 0.120	0.357
PT-LL	− 0.671	*0.000*	− 0.497	*0.001*	0.027	0.839
PT-TLK	0.126	0.539	0.179	0.257	− 0.010	0.940
PT-TK	− 0.458	*0.019*	− 0.419	*0.006*	0.274	*0.033*
PT-SVA	0.297	0.140	0.308	*0.047*	− 0.101	0.439
SS-LL	0.925	*0.000*	0.855	*0.000*	0.613	*0.000*
SS-TLK	0.211	0.301	0.242	0.122	0.017	0.897
SS-TK	0.534	*0.005*	0.661	*0.000*	0.169	0.193
SS-SVA	− 0.330	0.099	− 0.433	*0.004*	0.274	*0.033*
LL-TLK	0.056	0.788	0.109	0.492	− 0.068	0.603
LL-TK	0.721	*0.000*	0.770	*0.000*	0.209	0.106
LL-SVA	− 0.444	*0.023*	− 0.646	*0.000*	− 0.111	0.393
TLK-TK	− 0.135	0.510	− 0.141	0.373	0.222	0.086
TKL-SVA	− 0.025	0.902	− 0.140	0.377	0.301	*0.018*
TK-SVA	− 0.085	0.680	− 0.437	*0.004*	0.143	0.207

*LDH* lumbar disc herniation, *TK* thoracic kyphosis, *TLK* thoraco-lumbar junction kyphosis, *LL* lumbar lordosis, *PI* pelvic incidence, *SS* sacral slope, *PT* pelvic tilt, *SVA* sagittal vertical axis. *P values* (≤0.05) were considered statistically significant.

## Discussion

LDH patients sometimes have a forward-bending posture when walking. Spinopelvic alignment of the general population and LDH patients has been analyzed in some studies [11]. The scoliosis in LDH patients has also been studied in a few investigations [1–3]. However, the relationship of spinopelvic alignment between LDH patients with scoliosis and those without scoliosis has not been quantified at length.

The present study demonstrated that LDH patients and controls were significantly different in the anterior translation of the C7 plumb line, loss of LL and decrease in SS and TK and TLK. The loss of LL in LDH is not likely to be due to severe structural deformity. Rather, it may be secondary to segmental discopathy or small loss of disc height, a postural change secondary to an analgesic response to avoid posterior disc hyperpression, or foraminal stenosis due to a herniated disc [12]. The observed lumbar lordotic changes might also correspond to a rotation of the pelvis around the coxofemoral joints by contraction of the hip extensor muscles [13]. Biomechanically, the loss of LL would result in anterior displacement of the SVA as well as a more vertical PT to compensate for the anterior translation of the gravitational axis [14]. The correlations among parameters found in this study concord with that conclusion.

On the other hand, the PI in LDH patients in the present study was not significantly different from the control, a finding similar to the results of other studies [12, 15]. Previous studies suggested the association between the PI and lumbar degenerative diseases [12–14]. The smaller PI angle sometimes appears with the onset of certain lumbar degenerative disease. It is possible that the mechanism of LDH is different from other lumbar degenerative diseases.

In summary, LL loss, PT increase and the abnormal translation of SVA are possibly related to compensatory mechanisms that avoid increasing tension of the sciatic nerve in LDH patients.

The radiologic features of sciatic trunk shift were different from idiopathic scoliosis. Sciatic scoliosis exhibited a short lumbosacral curve accompanied by a long thoracic or thoracolumbar curve towards the opposite side and a relatively straight sagittal profile. The present results found that most patients with LDH had a small curve size that was associated with coronal imbalance. The main curve Cobb angle was 16.5°, the trunk shift was 2.6 cm, and 29.4% of patients showed a poor coronal balance. Notably, an association was observed between the side of the LDH and the direction of the scoliosis. A trunk shift of 50.0% of patients with LDH was towards the opposite side of disc herniation. A disc herniation location in 54.8% of patients was located on the concave side of the main curve. More than 80% of the patients showed deviation away from the painful side. It was hypothesized that the trunk was tilted laterally in response to irritation of the nerve root or hyperactivity of the paraspinal muscles. The appearance of patients with trunk shift was characterized as lateral and forward trunk list and lumbar hypolordosis. The present study compared values among patients with LDH, patients with trunk shift had lower SS and LL and higher SVA than did patients without trunk shift. Those may be explained as following: when LL decreases which result in pelvic retroversion, the centre of gravity will move backwards. However, to maintain balance, C7PL moves anteriorly more seriously to compensate for lower LL and higher PT. The loss of lordosis and a PT increase would lead to an anterior shift of the C7PL in LDH patients in patients accompanied with scoliosis.

The pathophysiology of trunk shift and scoliosis may be complex, and the compression of a nerve root by herniated disc material may not be a sole causative factor for sciatic trunk shift. Finneson [16] presumed that if the herniation is located medial to the nerve root, the scoliotic posture would be towards the side of the sciatica, and if the herniation is lateral to the nerve root, the scoliotic posture would go towards the opposite side of the sciatica. According to the present study, we put the preliminary hypothesis about scoliosis and trunk shift as follows: an unbalanced lumbo-pelvic muscular response to the irritation of the nerve root may result in trunk shift and scoliosis, and the asymmetry degeneration of the intervertebral disc may be an initiating factor which results in asymmetrical load on the spine at a segmental or even total spine, and then the vicious circle leads to scoliosis and trunk shift.

These abnormal parameters are mainly based on protective mechanisms designed to avoid sciatic pain. The abnormal translation of SVA and the abnormal posterior pelvic rotation are probably related to mechanisms that avoid increased tension of painful sciatic nerve. After surgery, hip extension followed by reduced sciatic nerve tension seems to allow recovery of SVA and pelvic anterior rotation [3].

Furthermore, the present study researched the relationship between coronal balance and disc herniation in LDH patients, which was not considered in detail by previous studies. The results showed no significant association between the side of LDH and the direction of trunk shift. Therefore, we consider that a trunk shift of patients with LDH is influenced by many factors, such as compensatory ability and lumbosacral curve.

## In conclusion

Sagittal spinal alignment of LDH with coronal trunk shift exhibits a more anterior shift of the C7 plumb line, less LL, and a more horizontal sacrum compared with that of controls and LDH without trunk shift. Correlation analysis showed a disharmonious spinopelvic relationship and a change in the compensatory model in patients with disc herniation. Therefore, the comprehension of spinal sagittal balance and morphotypes for specific situations may be conducive to earlier treatment possibilities and prevention measures.

## Data Availability

All data and materials were included in the article.

## Abbreviations

CSVL: Central sacral vertical line
CT: Computed tomography
LDH: Lumbar disc herniation
LL: Lumbar lordosis
MRI: Magnetic resonance imaging
PI: Pelvic incidence
PL: C7 plumb line
PT: Pelvic tilt
SS: Sacral slope
SVA: Sagittal vertical axis
TK: Thoracic kyphosis
TLK: Thoraco-lumbar junction kyphosis
